# Quantification of Blood Flow and Topology in Developing Vascular Networks

**DOI:** 10.1371/journal.pone.0096856

**Published:** 2014-05-13

**Authors:** Astrid Kloosterman, Beerend Hierck, Jerry Westerweel, Christian Poelma

**Affiliations:** 1 Laboratory for Aero & Hydrodynamics, Delft University of Technology, Delft, The Netherlands; 2 Department of Anatomy & Embryology, Leiden University Medical Center, Leiden, The Netherlands; University of Arizona, United States of America

## Abstract

Since fluid dynamics plays a critical role in vascular remodeling, quantification of the hemodynamics is crucial to gain more insight into this complex process. Better understanding of vascular development can improve prediction of the process, and may eventually even be used to influence the vascular structure. In this study, a methodology to quantify hemodynamics and network structure of developing vascular networks is described. The hemodynamic parameters and topology are derived from detailed local blood flow velocities, obtained by in vivo micro-PIV measurements. The use of such detailed flow measurements is shown to be essential, as blood vessels with a similar diameter can have a large variation in flow rate. Measurements are performed in the yolk sacs of seven chicken embryos at two developmental stages between HH 13+ and 17+. A large range of flow velocities (1 µm/s to 1 mm/s) is measured in blood vessels with diameters in the range of 25–500 µm. The quality of the data sets is investigated by verifying the flow balances in the branching points. This shows that the quality of the data sets of the seven embryos is comparable for all stages observed, and the data is suitable for further analysis with known accuracy. When comparing two subsequently characterized networks of the same embryo, vascular remodeling is observed in all seven networks. However, the character of remodeling in the seven embryos differs and can be non-intuitive, which confirms the necessity of quantification. To illustrate the potential of the data, we present a preliminary quantitative study of key network topology parameters and we compare these with theoretical design rules.

## Introduction

Fluid dynamics plays a critical role in the development of vascular networks [1]–[3]. Better understanding of vascular development can improve prediction of this process, and may eventually even be used to influence the final vascular structure. This can for example be useful in the fields of wound healing, drug delivery, or tumor control. To improve insight into the relation between hemodynamics and vascular development, quantification of the hemodynamics in the developing vascular structure is essential. Various models of the blood vessel structure and the influence of hemodynamics have been described [4]–[6]. Validation of such models is crucial for further application, and this requires experimental data. Some quantitative information on hemodynamics is already available for various types of vascular networks (e.g. [7]–[11]), as well as methods addressing difficulties resulting from incomplete experimental information in networks [12]. To study the hemodynamic and structural changes, this data should preferably be available for a large network, at more than one moment during development. Unfortunately, such data is currently still lacking. This study aims to address this by presenting a comprehensive data set, as well as the techniques used to obtain it.

The extra-embryonic vasculature of the chicken embryo (yolk sac vasculature or vitelline network) is a commonly-used model to study the development of the human cardiovascular system. During development, the network can be accessed multiple times provided that the embryo remains in the egg. This minimizes effects on the development of the embryo and vasculature. The long survival times in the *in ovo* studies reported by Korn [13] and the low survival rates in case of *ex ovo* development support this approach [14]. In addition, the *ex ovo* method may lead to shape changes of the network and thus altered mechanical stress distributions. As manipulation of embryo and vasculature is not required for the *in ovo* method, these detrimental effects can be avoided.

The vitelline network floats on top of the yolk sac. It starts as a collection of blood islands [15]. These consist of hematopoietic cells (primitive blood cells), surrounded by endothelial cells, which will later form the vessel walls. These blood islands align and will form a primary vascular plexus, also called the capillary plexus, consisting of small blood vessels. As soon as the heart starts beating, around HH stage 10 [16], blood starts flowing through the early embryonic vasculature and remodeling starts [15]. Remodeling of the capillary plexus starts just outside the body. From this point, remodeling extends further outwards, and the capillary plexus develops into a hierarchical tree. Eventually this will develop into distinct arterial and venous circulations [2].

Hemodynamic characterization at more than one instance requires non-invasive measurements of the local blood flow velocities in a given region of interest. It has been demonstrated that this can be achieved by *in vivo* microscopic Particle Image Velocimetry (micro-PIV). This technique has previously been applied to measure flow in the embryonic chicken heart [17], [18], as well as in the vitelline network [19], [20]. Micro-PIV is capable of obtaining a velocity field in two directions (perpendicular to the viewing direction) on a two-dimensional grid in a planar domain [21]. This is sufficient to describe the hemodynamics in the network: during the early developmental stages investigated in this study the vitelline network is predominantly confined to a thin planar domain. Furthermore, as the flow is laminar, the two in-plane velocity components will be sufficient to describe the flow in these blood vessels.

PIV is based on the displacements of particles in the flow, either artificial or naturally present. First, a series of image pairs is recorded to capture these particles. For the analysis, the measurement area is divided into smaller subdomains, called interrogation areas. These define the spatial resolution of the result. Cross-correlation is applied on the interrogation windows to obtain a local displacement, and eventually a local velocity. This statistical approach, determining the most likely displacement for a group of tracer particles, makes PIV a more robust method compared to other measurement techniques based on tracking of individual particles. A more extensive description of PIV can be found in the literature [21].

**Figure 1:**
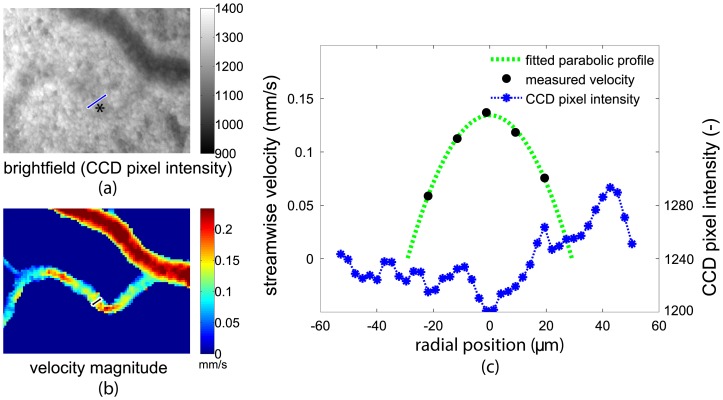
Detecting the vessel wall from both the brightfield images and the fitted velocity profile. The measured streamwise velocity (black dots) with fitted parabolic velocity profile (green dotted line) and the corresponding pixel intensity values of the CCD (blue) are shown for a typical cross section of a vessel. The pixel intensity values are averaged over 50 recorded images to improve the signal to noise ratio. The brightfield image and corresponding time-averaged velocity magnitude are shown on the left, with the locations of the cross sections in blue and black, respectively.

PIV not only provides local velocity data, but can also be used to obtain the network topology (see Section *Methods*). In principle, this topology could also be obtained from the brightfield images, which are recorded as part of the PIV measurements. However, the imaging conditions sometimes do not allow a robust detection of smaller blood vessels, see Figure 1. The wall location of the large blood vessel can be obtained by thresholding the intensity of the bright-field image (Figure 1a). However, the smaller blood vessel (indicated by *) is hardly distinguishable in the same figure. In contrast, the velocity magnitude field, as obtained by PIV, provides a much more distinct structure. This is illustrated in Figure 1c, which shows the local velocity (black dots) along the cross-section indicated in the figure, along with the fitted parabolic velocity profile (green dotted line). The corresponding pixel intensity values of the CCD (blue) are also shown. As can be seen in the figure, the vessel walls can easily be detected using the parabolic fitted velocity profile, but detecting the walls based on the pixel intensity is not straightforward.

In this study, we make use of the capabilities of *in vivo* micro-PIV to quantify hemodynamics and determine the network structure in a vitelline network. Moreover, this is performed multiple times at developmental stages between HH 13+ and 17+ (2–3 day old). At HH stage 13+ remodeling has started, and significant changes in the network structure take place in progression through the subsequent stages. During the later stages (after HH 17+), the two distinct arterial and venous networks start to extend into three-dimensions. These stages are therefore excluded in this study. We focus on stages HH 13–17 and report data obtained from seven chicken embryos at two developmental stages. We evaluate the accuracy of the method and present a preliminary analysis of the data set to show its potential to study both local and global changes in the vasculature. Examples of the former are the fate of individual vessels, while the latter refers to a more statistical approach of the entire vasculature.

## Methods

### Chicken Embryos

**Figure 2:**
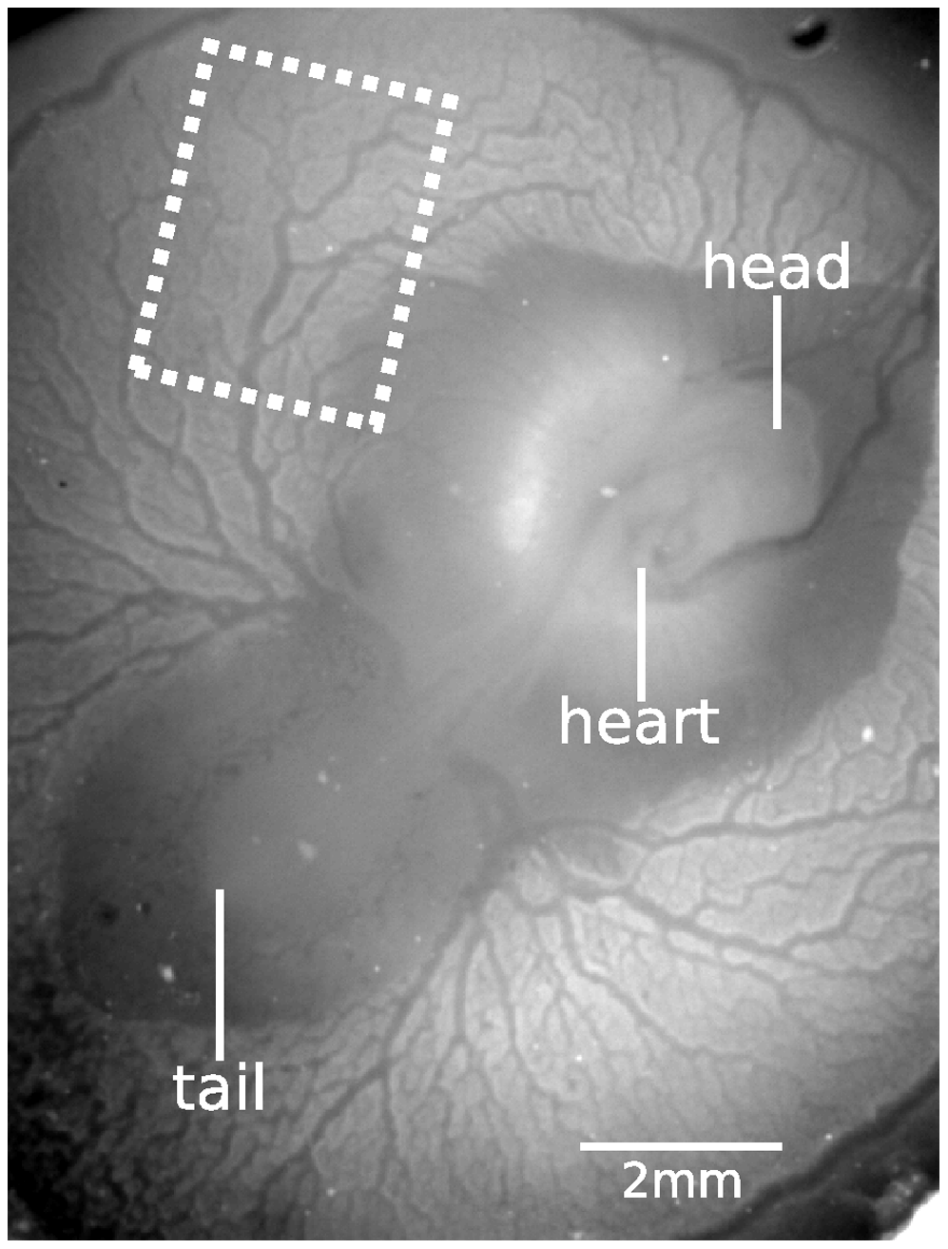
The embryo and vitelline network during the third day after fertilization are clearly visible after windowing the egg. A typical measurement region is indicated by the dashed white box.

Fertilized White Leghorn eggs were incubated for 60 to 77 hours at a constant temperature of 38°C and a constant humidity. During this incubation time, they remained in the same position, lying horizontally. As a consequence, the chicken embryo and the surrounding vitelline network were floating on top of the egg yolk. To perform *in vivo* micro-PIV measurements, optical access to the embryo and the vitelline network was required. For this purpose, a viewing window was carefully created in the egg shell [13]. The size of this window should approximately match the size of the network to enable inspection for irregularities of the entire network, and thus measured a few square centimeters. To prevent dehydration of the embryo and the vitelline network, Locke saline solution (0.94% NaCl, 0.0045% KCl, 0.004% CaCl_2_ (w/v)) was added. To enhance the image quality, the window was then covered with a microscope cover glass before placing it under the microscope. The embryo and vitelline network were first checked for irregularities, and embryos with abnormal development (for example embryos lying on their right side or embryos with visible network damage) were excluded from the study. Preceding the velocity measurements, heart rates were measured to ensure a normal heart rate, and the developmental stages of the embryos [16] were determined (see Table 1). The stability of the heart rate throughout the measurement was evaluated *a posteriori* by analyzing the instantaneous velocity at the centerline of a large artery and counting local maxima. The heart rates during the measurement series are also shown in Table 1. Measurements using micro-PIV were performed based on bright-field imaging, as described in the next section. An overview image of a chicken embryo and surrounding vitelline network with a typical measurement region is shown in Figure 2. A large group of 95 embryos were incubated for this study, but useful results were obtained for only 7 embryos. This small number is mainly due to the short time where the embryos have the desired developmental stages. Furthermore, the experiments required a correct position of the embryo and vitelline network within the shell window throughout the measurements.

**Table 1:** Measurement information for every embryo.

embryo	HH stage		HR (BPM)		*A* (mm^2^)	vessel segments (−)(quantified) (−)		vessel occupancy(−)		vessel density (1/mm^2^)	
label (no.)	T1	T2	T1	T2		T1	T2	T1	T2	T1	T2
1	13+	14+	66	76	3×5.5	455(368)	352(259)	0.36	0.31	29	20
2	14	16	48	80	1.5×3.5	102(88)	94(85)	0.32	0.27	21	20
3	14	16	81	78	3.5×4.5	172(126)	265(228)	0.22	0.26	11	18
4	14	16	105	95	3×6	288(255)	270(236)	0.29	0.27	17	15
5	15	16	97	109	3×4.5	224(189)	491(386)	0.33	0.39	18	34
6	15	16	61	75	3.5×3	299(252)	431(349)	0.37	0.44	32	40
7	16	17+	106	107	3×4	645(510)	680(570)	0.33	0.40	40	49

Measurement information for every embryo, for both measurement series (T1 and T2): developmental HH stage, heart rate HR (beats per minute), measurement region surface *A* (mm^2^), number of vessel segments present in the measurement region (followed by: number of vessel segments present in the measurement region characterized by corresponding velocity and diameter), vessel occupancy (average relative area occupied by vessel segments), and vessel density (average number of vessel segments/mm^2^).

Following the first micro-PIV measurement series, the egg was placed back into the incubator for further development, after having covered the window in the egg with clear plastic tape to protect the embryo from dehydration. Before this, a few millilitres of albumen was carefully removed through the small hole in the base of the egg to make sure the embryo and vitelline network would not stick to the plastic tape during their stay in the incubator. When necessary, additional Locke saline solution was dripped onto the egg during the remaining incubation time. For the second measurement series, 3 to 9 hours later (see Table 1), the plastic tape covering the window was removed and the egg was again filled up with Locke saline solution and covered with a microscope cover glass.

### Measurement Setup

**Figure 3:**
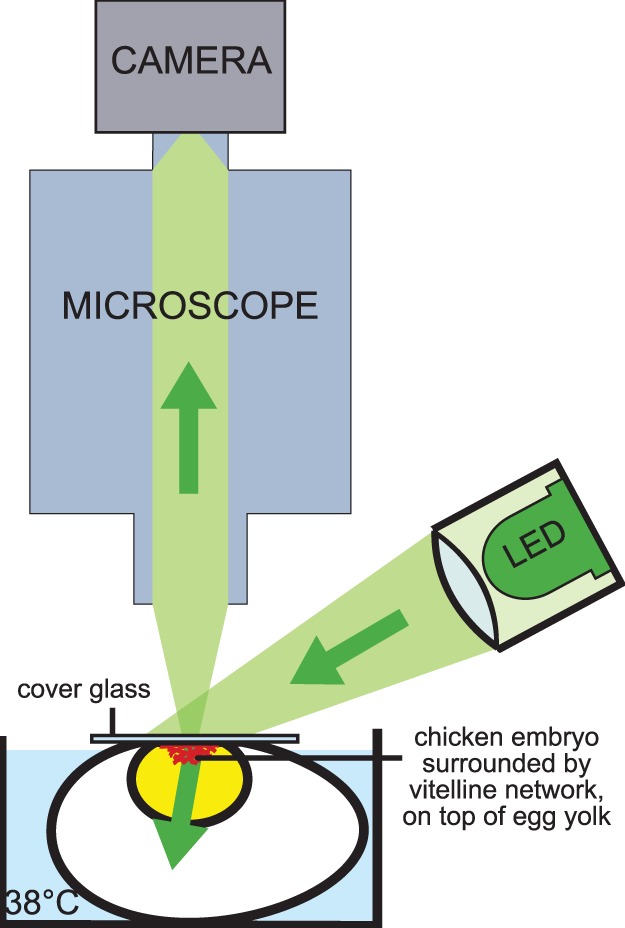
For the micro-PIV measurements in the vitelline network, the embryo is placed in a bath of constant temperature of 38°C under the microscope. The vitelline network is clearly visible through the microscope since the network and embryo are floating on top of the egg yolk. A high-powered LED illuminates the tissue directly, not through the microscope.

For the micro-PIV measurements, the egg was placed under a microscope in a bath containing water with a constant temperature of 38°C to ensure constant temperature and heart rate during the measurements (see Figure 3). An upright epifluorescent combi-microscope with zoom functionality (Leica MZ 16 FA) was used with a Leica FluoCombi III objective, providing magnifications in the range of 2.8–46×. A magnification of 5× with corresponding numerical aperture 0.14 was used. This magnification was chosen such that the resulting measurement resolution is sufficiently high while the measurement time remains acceptably small (up to 30 minutes for one measurement series, see the next section) compared to the time in which development takes place. A PCO Sensicam QE camera (1,376×1,040 pixels) was used for image recording, with 2×2 binning to improve the signal to noise ratio. This also enabled a two times higher acquisition rate, which shortened the time needed for the acquisition of the images and ensured that the pulsatile flow was adequately sampled. The resulting pixel size was 12.9 × 12.9 *µ*m^2^, and one individual measurement area thus covered 1.8×1.4 mm^2^. The dimensions of the combined measurement regions are given in Table 1.

**Figure 4:**
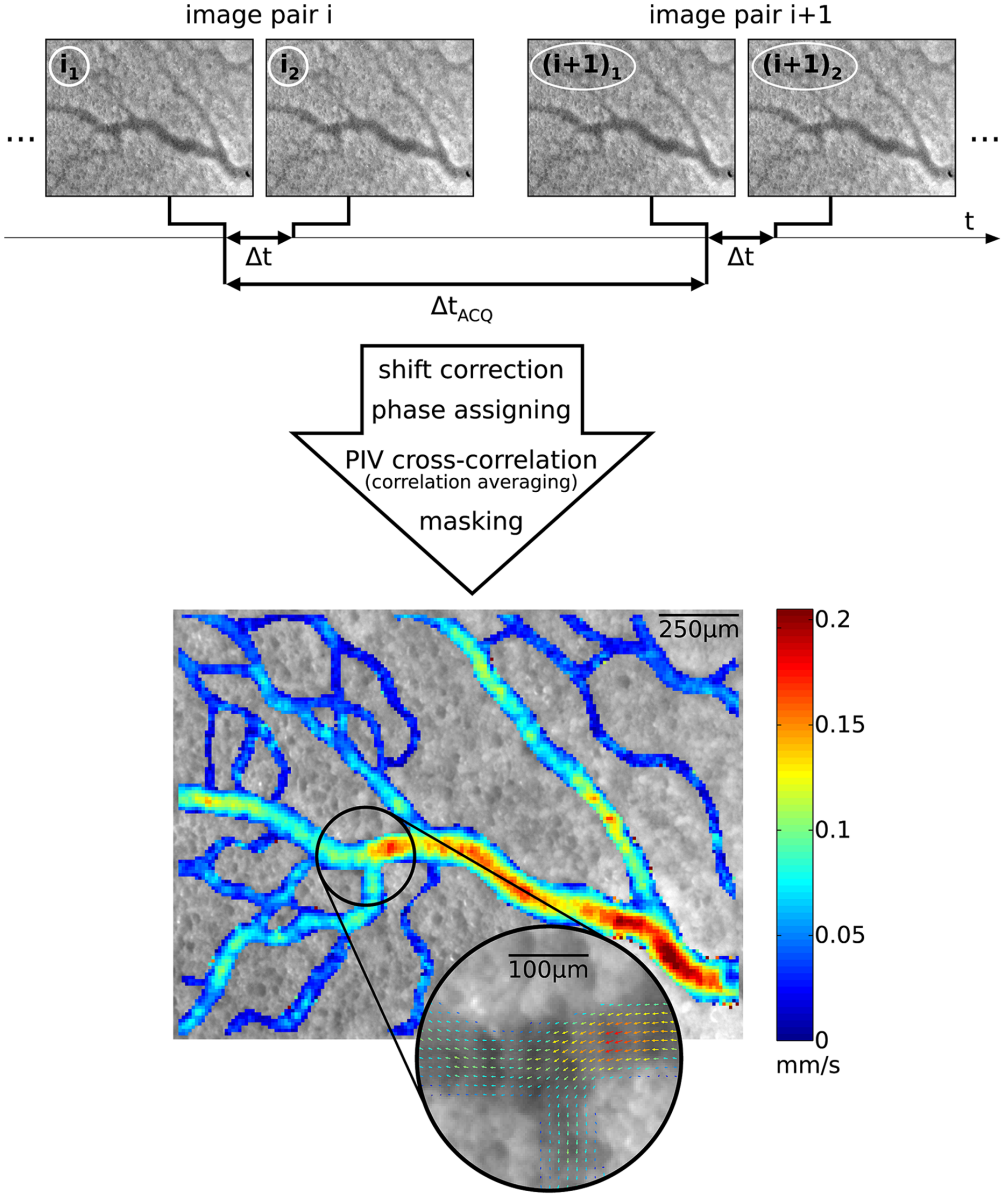
Subsequent image pairs are recorded to obtain a velocity field. After applying several processing steps, a two-dimensional velocity vector field yielding two velocity components is obtained. One measurement area is shown (embryo 3, second measurement series; T2), displaying the time-averaged velocity magnitude, 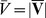. A selection is enlarged to show the corresponding time-averaged velocity vector field, illustrating the spatial resolution of the measurement results.

Red blood cells (RBCs) were used as tracer, since adding artificially tracer particles was less suitable for repeated measurements spanning several hours [22]. The larger size of RBCs (compared to typical artificial tracers) leads to an increased depth of correlation (DOC), which defines the measurement plane thickness [23]. A relatively large DOC compared to the blood vessel diameter can cause a severe underestimation of the measured centerline velocity (see [24]), since the measured velocity is an average of all flow velocities along the viewing direction. With the present magnification of 5× and the largest blood vessels diameter measuring 200 *µ*m, the measurements were in the depth-saturated regime [20]. In this case, the actual centerline velocity could easily be retrieved by applying a correction factor. Moreover, the measured velocities in the depth-saturated regime are less sensitive to small misalignments of the measurement plane with respect to the centerlines of the vessels compared to measurements using a relatively small DOC. A pulsed high-powered light emitting diode (LED; ILA GmbH) was used as light source to image the RBCs [20], [25]. They appeared as black dots in the recorded images (see Figure 4).

### Data Acquisition and Processing

Double-frame recording was used for the *in vivo* micro-PIV measurements using DaVis software (LaVision; Göttingen Germany), see Figure 4. This entails that subsequent image *pairs* were recorded, at a frequency of 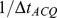. Each image pair consisted of two recordings on separate frames, with a time difference of 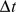. The recording frequency, or repetition rate, 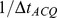 was set to its maximum, 9.7 Hz. This was independent of the heart rate of the chicken embryo, and hence image pairs were recorded on multiple and arbitrary moments during several cardiac cycles. The time difference between two frames of one image pair, 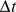, was chosen such that the maximum displacement occurring in all areas of one measurement region would not exceed 10–15 pixels during the cardiac cycle. As the peak velocities varied throughout the network and also varied with age, 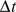 was determined for every measurement series separately. The maximum displacement restriction was achieved with 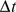 = 10 ms, and sometimes 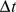 = 5 ms or 20 ms. In some measurement regions the dynamic range of blood flow exceeded the measurable range, for instance if both very large and small blood vessels were present in the same field-of-view. In these cases, the velocities were also determined using first-frame correlation (see Figure 4). In this approach velocities were not based on correlating the two frames of one *pair* (i_1_ and i_2_), but by correlating the *first frames* of two subsequent pairs (i_1_ and (i+1)_1_). As the temporal delay between the two first frames is much larger than the 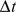 between the frames of an image pair, this provides a much larger dynamic range. By combining both correlation approaches, the dynamic range was enlarged from 0.01–1.2 mm/s to 0.001–1.2 mm/s [22].

For each measurement area, 500 image pairs were recorded, which took less than one minute. This number was sufficient to apply correlation averaging [26] in order to obtain ten phase-locked velocity field during the cardiac cycle [22]. Correlation averaging based on 50 image pairs significantly improved the accuracy of each phase-locked velocity field, while the total recording time remained acceptable. After recording the 500 image pairs, the embryo was translated using a translation stage to obtain adjacent regions; typically 9 areas were documented per embryo and stage.

The PIV data of multiple adjacent measurement areas, recorded in a single measurement series, were processed and combined to obtain one measurement result with the time-averaged velocity field at one developmental stage. The image data were processed using an in-house multi-pass PIV code [19], with a final interrogation area size of 8×8 pixels using 50% overlap. The full details of this processing are described in detail elsewhere [22]. It entailed image drift correction, ‘stitching’ of the pulsatile velocity fields and temporal averaging. The latter was done here to reduce the amount of data, but transient results are readily available. Averaging had to be done with care at the appropriate step during the processing [18]: for strongly pulsatile flows the image data had to be sorted first based on the phase within the cardiac cycle; after processing, the resulting vector fields could be averaged. For weakly pulsatile flows the raw images could be processed without sorting. The velocity fields were then masked to exclude regions with outliers (generally occurring outside the blood vessels) by a median filter. The end result of the PIV analysis and post-processing was, for each embryo and stage, a time-averaged velocity field of typically 3×5 mm^2^ with a relatively high spatial resolution of 10×10 *μ*m^2^.

### Hemodynamic Parameter Extraction

To facilitate a quantitative analysis, each measured two-dimensional velocity field was modeled as a collection of connected vessel segments. Vessel segments were each characterized by a single diameter, velocity and length. Diameter and velocity were obtained by averaging results of multiple adjacent extracted blood vessel velocity profiles. Based on these parameters, other relevant hemodynamic parameters can be calculated, such as e.g. the flow rate and the wall shear stress. Typical values for embryonic avian blood properties, required for the determination of the wall shear stress, are reported in the literature [27].

**Figure 5:**
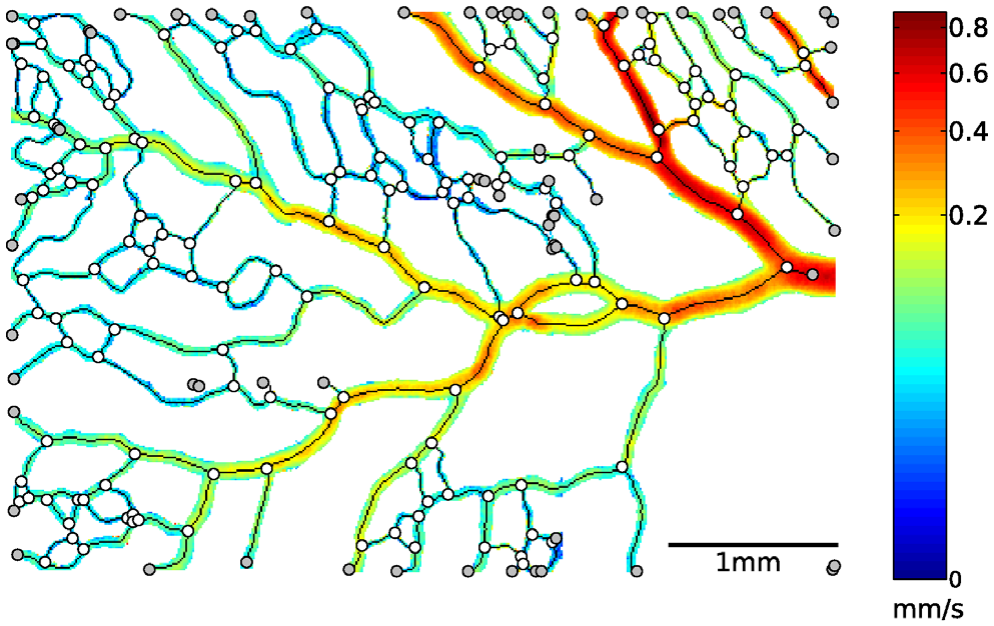
Time-averaged velocity magnitudes of a measurement region are shown, together with with the corresponding skeleton (black line), branch points (white circles), and end points (gray circles). The flow enters the measurement region from the right. Note that a non-linear color scale was used for the velocity magnitude.

To define the network structure, blood vessel branches were first automatically identified based on the skeleton [28], [29]. Vessel segments between two branch points were subsequently labeled using a Matlab routine [22], [30]. As an illustration, a measurement region with corresponding time-averaged velocity magnitudes is shown in Figure 5. The non-linear color-coding was adapted such that all velocity scales are visualized. The skeleton is displayed as a black line, branch points are marked by white circles, and end points are marked by gray circles. Accordingly, the skeleton’s part between two nearest branch points (including end points) approximately coincides with the centerline of one vessel segment. This way, the collection of vessel segments and their connectivity was defined.

**Figure 6:**
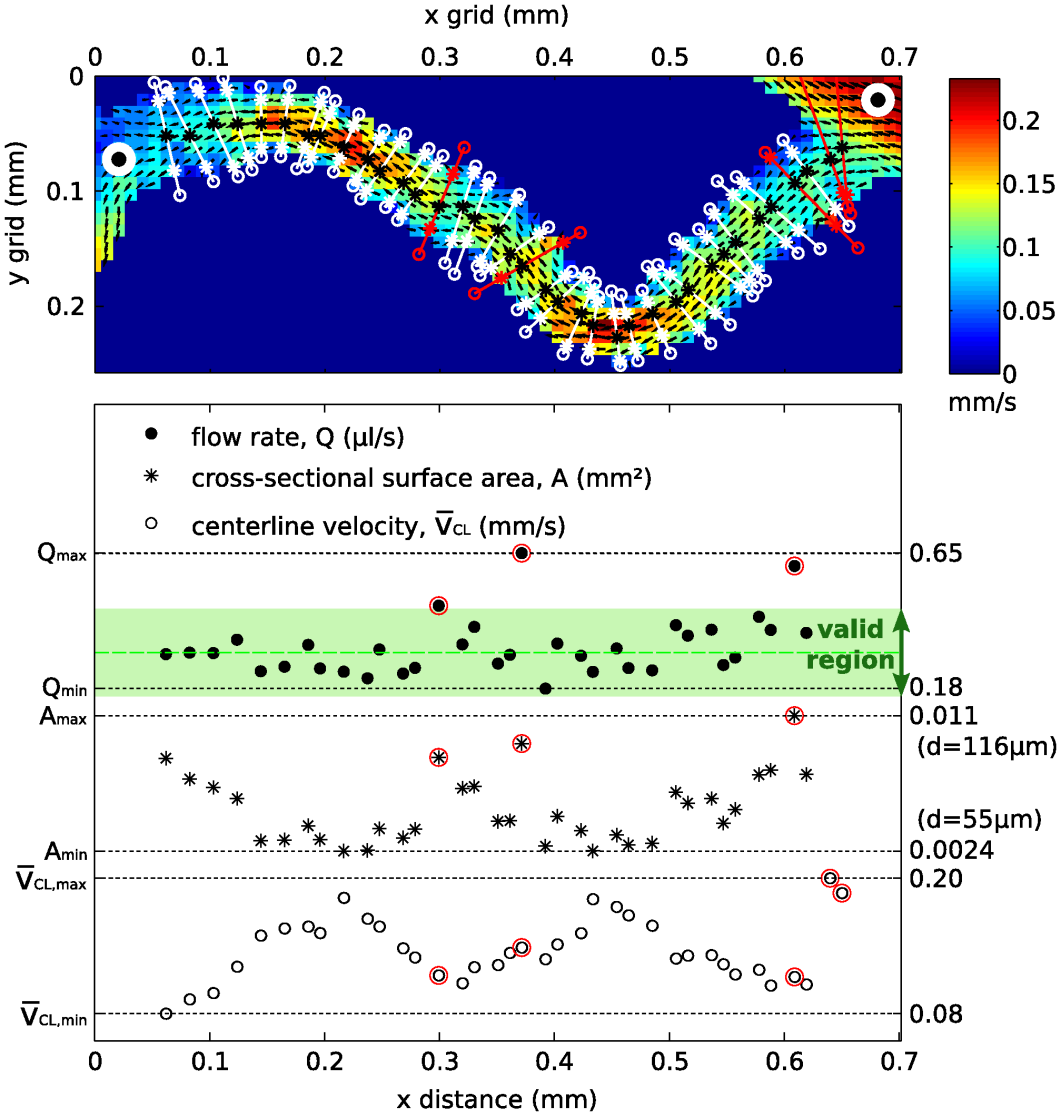
Multiple parabolic fits at several locations on the vessel centerline were performed to obtain a single characteristic velocity and diameter for each vessel segment. The time-averaged flow rate is assumed constant throughout the vessel segment. The valid region is bounded by 0.5 and 1.5×the median flow rate, and the red-encircled data points were not incorporated, due to a strongly deviating flow rate. Note that the fitted diameters and flow rates for the two data points on the far right are too large to be visible in the graph.

Next, a characteristic velocity and diameter for each vessel segment were extracted. The procedure is explained using one vessel segment, shown in the top part of Figure 6. An extensive description of the procedure is given by Kloosterman [22]. To obtain a single characteristic vessel diameter and velocity, the flow rates were assumed to remain constant throughout each vessel segment. The entrance length of the flow can be considered to be small compared to the length of a vessel segment due to the low Reynolds number (Re<<1), and the flow could therefore be considered fully-developed throughout each vessel segment. A polynomial fit was used to obtain vessel diameter and centerline velocity. At least three points are needed for a polynomial fit. This limited our approach to blood vessels larger than three times the measurement resolution, i.e. approximately 30 *μ*m.

Poelma et al. (2010) [18] showed that the approach to determine the wall location based on the velocity profile could be accurate within a few micrometers in a comparable *in vitro* experiment. Avian blood has a relatively constant viscosity, in contrast to the well-known strongly shear-thinning behavior of human blood [27]. This implies that the velocity profile will be nearly parabolic, which has indeed been confirmed in previous experiments [20]. At multiple cross sections of a vessel segment (white and red lines in the top graph), parabolas (second-order polynomial) were fitted to the local velocity profiles, which gave local centerline velocities (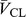) and diameters *d*. Neighbourhoods of branch points were omitted in this procedure since defining the streamwise direction here is not straightforward. The trend showed that the centerline velocity (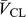, on the bottom of the graph) was inversely proportional to the cross-sectional surface area (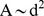, middle part of the graph). An average flow rate *Q* was determined based on the valid flow rates (flow rates between 0.5 and 1.5× the median flow rate). These data points are located in the figure in the green valid region; excluded data points are encircled red. The standard deviation of the valid flow rates was generally an order of magnitude smaller than the average flow rate. Now, the characteristic centerline velocity was determined by averaging the velocities from the valid data points. The characteristic diameter was subsequently derived from the average flow rate and characteristic centerline velocity. Additionally, the result was visually inspected to detect any remaining anomalies that might still be present.

### Data Quality Assessment

It was not possible to directly validate the measurement technique *in vivo*. In an *in vitro* validation experiment, the technique has previously been shown to be capable of measuring flow rates with an error less than 2% error [18]. For the current *in vivo* application, the only way to assess the accuracy of the velocity measurements and the subsequent segment identification was to perform internal checks. An example is the aforementioned variation of the flow rate in a single vessel, which can only be due to measurement errors. To compare the performance of the parameter extraction method, the cross-sectional velocities and corresponding fitted velocity profiles for three vessel segments were investigated in detail. These segments were selected to have a similar diameter, but with significantly different velocities.

The quality of the total data set was also investigated by verifying the flow balances in all branch points [31]. The flow balance is defined by 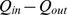, and the relative flow balance by 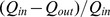, where 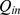 and 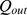 is the total flow entering and leaving the branch point, respectively. An ideal flow balance without measurement errors is obviously established when 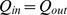. For every measurement region the distribution of flow balances was studied. A relative small number of large outliers, where 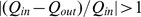, was excluded.

Our method is based on representing the flow based on a polynomial fit. Measurement errors are more likely to occur near the wall, where the velocity gradients are at a maximum. Furthermore, fits in larger vessels are based on more velocity information. The uncertainty in the fit - and thus flow rate - can therefore be expected to decrease for increasing vessel diameters. To check this, we evaluated the flow balances for both the full collection of blood vessels, as well as a subset containing only the larger vessels. These were identified as vessels larger than 52 *μ*m (5 times the spatial resolution of the velocity measurements). This subset was roughly three-quarters of the whole set of vessels.

### Structural Parameters

**Figure 7:**
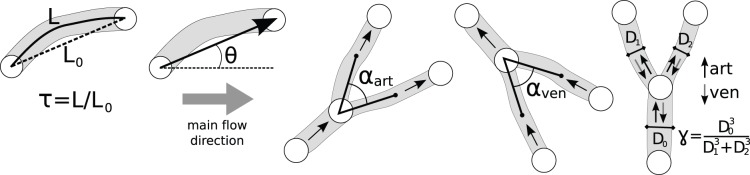
Schematic diagrams of studied structural parameters are shown for clarity. These parameters are: the tortuosity 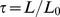, the angle between the vessel segment and the main flow direction 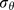, the arterial branching angle 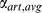, the venous branching angle 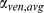, and Murray’s law ratio for both arterial and venous branches 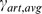 and 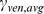.

In this study, six additional parameters were calculated to study structural changes between two consecutive networks. The first two parameters were related to the total network structure: the vessel occupancy and the vessel density. These were defined as the part of a measurement region occupied by vessel segments, and the average number of vessel segments present in one square millimeter. The tortuosity 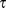, defined as the ratio of the actual path length *L* and the Euclidean distance between the two adjacent nodes 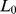, as well as the angular vessel flow 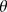, defined as the angle between the main flow direction in the total measurement region and the local vessel flow direction (see Figure 7 for illustrations), were determined for all vessel segments.

Also the bifurcations were inspected. Parameters derived from design principles for optimal networks were validated by the experimental values from this study. Every branch point connects one parent vessel with two child vessels having the same flow direction: towards (converging, in this study defined as venous) or away from (diverging, in this study defined as arterial) the branch point. Note that the arterial and venous bifurcations were defined in this study by their flow directions (diverging or converging, respectively), and not by specific gene expression. First, the branching angle 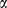 was determined. This was defined by the angle between the two lines connecting the branch point with respectively the center of one daughter vessel halfway the two adjacent branch points, and the center of the other daughter vessel halfway the two adjacent branch points (see Figure 7 for schematic diagrams).

Much research has been done on the validation of Murray’s law ([32]–[34], among others), also in the vitelline network of the chicken embryo [35], [36]. The design principle of a minimal cost function leading to this law was first introduced by Murray in 1926 [37], [38], and holds the relation between the parent vessel diameter 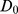 and child vessel diameters 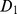 and 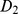: 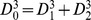. To validate this law for our data sets, the parameter 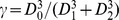 was computed, and should ideally be equal to one. Since this parameter is very sensitive to outliers, the impact of outliers was reduced by removing the largest outlier from the data set (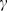 exceeding the value of three). The value of three was high enough to exclude the large outliers affecting the statistics; lowering this limit had a minimal effect since only a few outliers were removed.

## Results and Discussion

The vitelline networks of the seven embryos at two consecutive developmental stages (T1 and T2) included in the study were modeled as a collection of connected vessel segments, each with one characteristic diameter, velocity, and length. The complete collection of modeled networks of all seven embryos can be found in Appendix B in Ref. [22]. The data (File S1) and visualizations (Figure S1) of all networks are also available as electronic supplementary material of this manuscript.

### Data Quality

**Figure 8:**
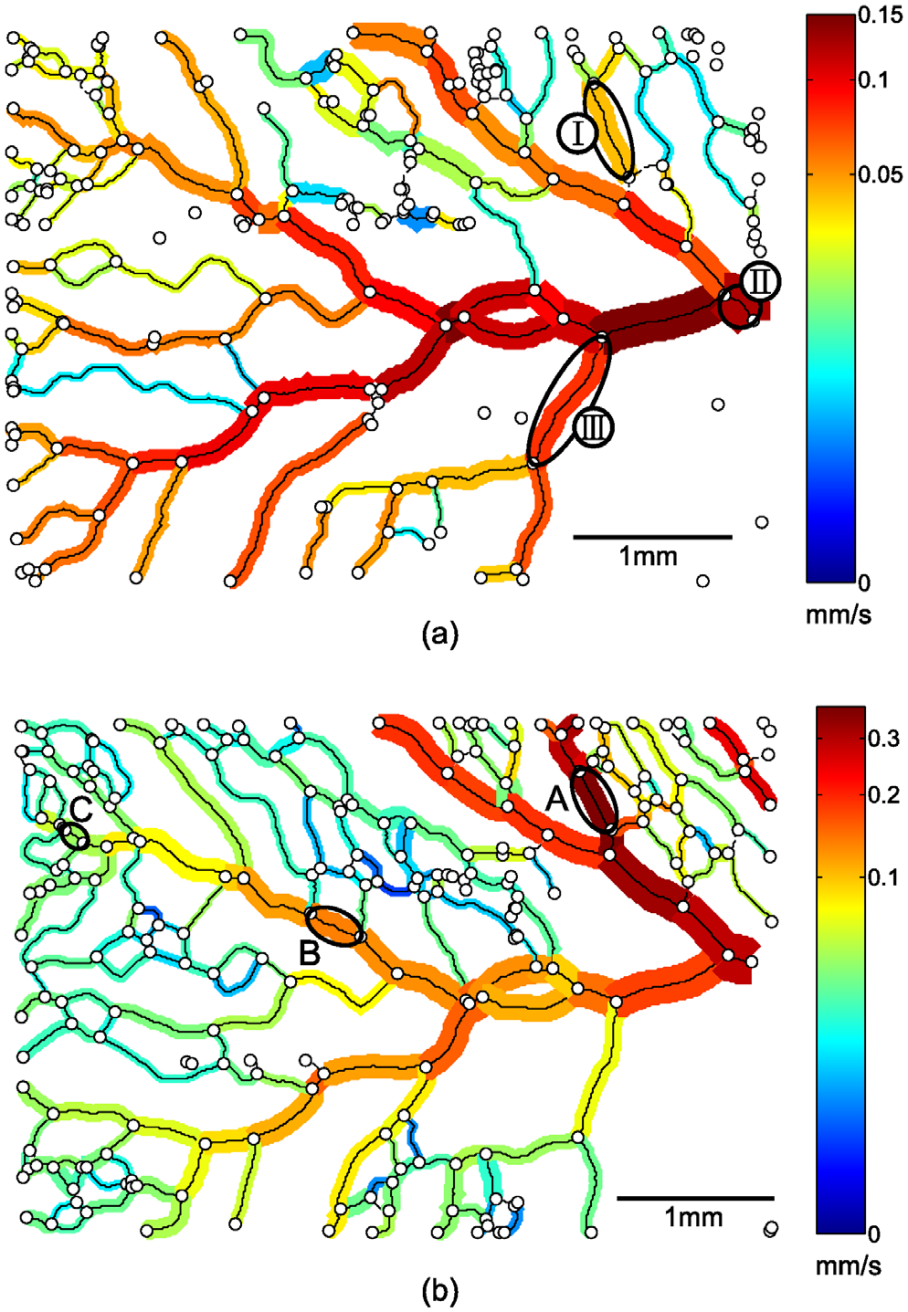
Networks are modeled as a collection of connected vessel segments, here shown with corresponding diameter and color-coded time-averaged mean velocity for two consecutive measurements T1(a) and T2(b). Besides a general velocity increase, the part of the network in the upper right corner seems to have become responsible for a larger part of the total blood flow in this region (see also text). The flow enters the measurement region from the right. Note the two different non-linear color scales for (a) and (b).

**Figure 9:**
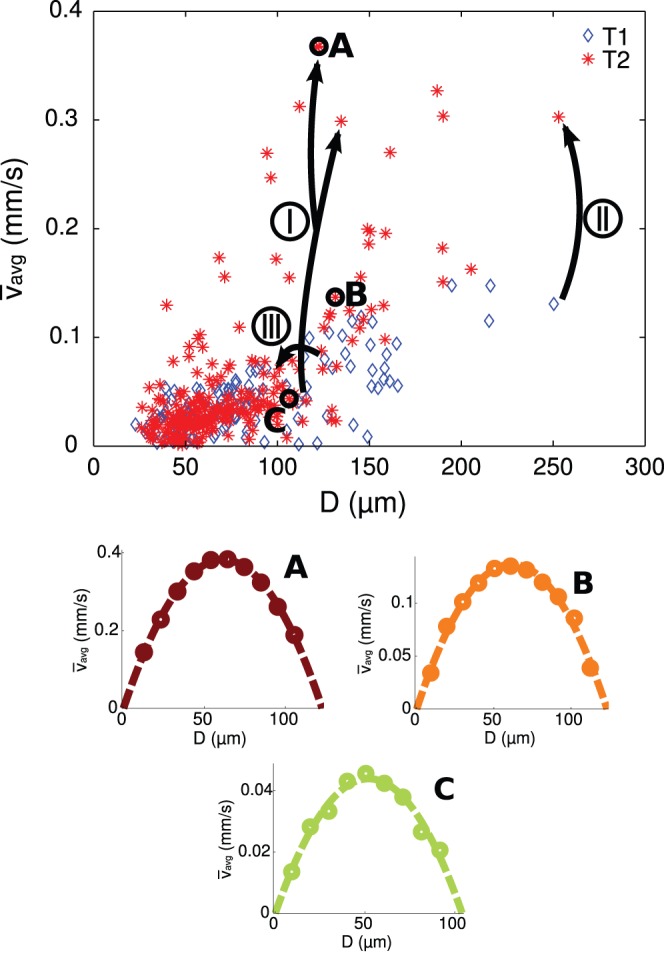
The variety in time-averaged mean velocities for all vessel segment diameters are shown for two consecutive measurements T1 and T2. For three vessel segments (I, II, and III, indicated in Figure 8), the changes in diameter and time-averaged velocity from T1 tot T2 are indicated by the dashed arrows. The cross-sectional velocity profiles shown for three data points (A, B, and C, indicated in Figure 8) show consistency despite the different time-averaged velocities.

For the three vessel segments A, B, and C in Figure 8, the cross-sectional measured velocities and corresponding fitted velocity profiles are shown in Figure 9. The diameters are in the same range: between 106 and 131 *µ*m. The velocity differences, however, are considerably larger: the velocity in vessel segment A is almost ten times the velocity in vessel segment C, but a clear parabolic shape of the cross-sectional velocity is observed for all three vessel segments. The comparable performance of the method in a large velocity range clearly indicates that the variation in the extracted velocity in one measurement region is caused by biological variance in dimensions and flow, and is not a consequence of inaccurate measurements or fitting procedures. This also implies that estimating local blood velocities from vessel diameter (for example by Pries et al. (1990) [39]) may lead to errors; as can be seen in Figure 9, a vessel with a diameter of 100 *µ*m can have a centerline velocity that varies over an order of magnitude.

**Figure 10:**
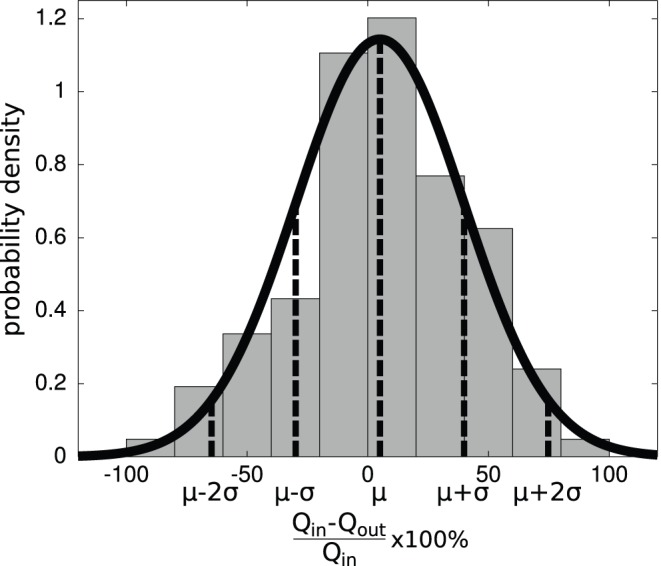
The bar chart shows the probability density function of the relative flow balance 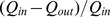 for embryo 3, at T2. This can be represented by a normal distribution which should ideally have a mean 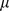 close to zero and a small standard deviation 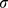.

The distribution of the relative flow balance of each measurement region could be approximated by a normal distribution, when excluding a relative small number of large outliers where 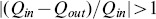. The bar chart in Figure 10 shows the probability density function of the relative flow balance for embryo 3 at T2; the thick black curve represents the fitted normal distribution. Additionally, the corresponding mean 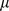 and boundaries of the respectively 68% and 95% tolerance intervals (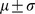 and 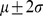) are indicated. According to these parameters, 68% of the branch points have a relative flow balance between −30% and 40% for this measurement region. A summary of the mean, standard deviations, and percentage of outliers can be found in Table 2 (listed under ‘all diameters’). The mean values are close to the theoretical value of zero for all embryos, so there appears to be no systematic flow measurement error. Comparing the statistics for the whole set and the ‘large diameter’ subset, there seems to be no difference. This suggests that the relatively larger errors in smaller vessels (as anticipated in the Section *Data Quality Assessment*) appear to be absent. Note that the standard deviations of the relative flow balance 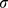 reported are significantly larger than the standard deviations of the flows *Q* that are used to calculate the balance. Error progression can easily double the error in the reported values for 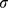. This means that a conservative estimate of the standard deviation of the flow *Q* is approximately 20%; this is somewhat higher than the variation along the centerline reported earlier (see e.g. Figure 6).

**Table 2:** Statistics of the relative flow balance for every embryo.

embryo	T1				T2			
	all diameters		large diameters		all diameters		large diameters	
label (no.)	*μ*±*σ*	Outliers (%)	*μ*±*σ*	Outliers (%)	*μ*±*σ*	outliers (%)	*μ*±*σ*	Outliers (%)
1	8±44	16	7±44	10	6±36	13	9±33	14
2	1±43	7	−5±32	0	0±40	0	−14±31	0
3	3±33	10	10±24	0	5±35	9	9±29	4
4	−1±31	4	−3±30	3	0±30	7	2±22	8
5	−4±28	2	1±21	0	2±31	5	1±24	2
6	1±35	4	2±34	2	4±33	11	2±31	9
7	3±41	11	2±37	7	8±45	14	5±46	21

The statistics of the relative flow balance 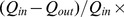
100% are presented for every embryo for both measurement series (T1 and T2): mean 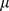, standard daviation 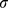, and the percentage of outliers holding 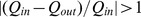. These parameters are presented for both the total available data set listed under ‘all diameters’, and the selected data set containing only vessel segments with diameters larger than 5 times the spatial resolution (corresponding to 52 *μ*m, listed under ‘large diameters’).

The two cases that deviate most from the theoretical situation and also have the largest relative numbers of outliers are the measurements of embryo 1 at T1 and embryo 7 at T2. For embryo 1, this can be explained by the fact that at the time of measurement, this embryos had only reached HH stage 13+. At this stage, the vitelline network still resembles a sponge-like structure [2], [40] and it is difficult to define individual blood vessels and thus network topology. For the case T2 of embryo 7, the embryo had reached HH stage 17+. At this stage, the network starts to extend into three-dimensions instead of being confined to a plane. This is due to the growth on the curved yolk sac. The measured velocities are hence a projection of the actual velocities onto the measurement plane, and are likely to be slightly underestimated. This also results in a less accurate overall flow balance.

### Example of Network Remodeling

Figure 8 shows the two representations for embryo 3. This embryo developed from HH stage 14 to 16 during this study, which took approximately 4 hours. Despite this apparently short time, significant differences in flow and structural changes occurred. The maximum time-averaged velocity in this measurement region more than doubled (with an even greater increase in volumetric flow rate). The preferred direction also seems to have changed: the part of the network in the upper right corner seems to have become responsible for a larger portion of the total blood flow. For this embryo, it means that the preferred direction of the blood flow shifted towards the tail of the embryo. The changing hemodynamics are also clearly visible in the upper part of Figure 9, where time-averaged mean (bulk) velocities are shown as function of the respective vessel segment diameter for the two stages T1 and T2. For three vessel segments (I, II, and III, indicated in the top part of Figure 8), changes in diameter and time-averaged velocity from T1 tot T2 are indicated by the dashed arrows. The development of these three vessel segments show completely different behavior. Where vessel segment I splits into two vessel segments due to a new branch midway along the segment, the time-averaged velocity and flow in vessel segment II more than doubled with minimal change in diameter. On the contrary, the time-averaged velocity and flow rate in vessel segment III decreased.

### Qualitative and Quantitative Observations

For every embryo in this study, changes in structure and hemodynamics took place between the first and second measurement series. Direct comparison between the embryos was difficult, since the measurement regions of the seven embryos were not always located in the same part of the vitelline network. However, one could conclude that remodeling took place in all developmental stages in this study, and a rich array of observed phenomena was observed. The qualitative changes were compared with the quantitative results, and are described in the following paragraphs.

The distributions of the characteristic diameters *D*, time-averaged velocities 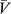, and lengths *L* of the vessel segments were represented by the 25%–50%–75% percentiles for all measurement series (see Table 3). During development, minimal changes in the percentiles of the diameters were observed (up to 23% difference in the percentiles). However, the overall velocity increased by a factor of three in some cases. This large velocity increase and a minimal heart rate increase were measured for embryo 7. Despite the fact that the mean velocity can be influenced by the heart rate, the changes in velocity due to development are found to be more substantial.

**Table 3:** Distributions of the characteristic hemodynamic parameters for every embryo.

embryo	percentiles *D* (*μ*m)		percentiles 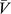 (*μ*m/s)		percentiles *L* (mm)	
label (no.)	T1	T2	T1	T2	T1	T2
1	56–82–119	52–76–119	25–46–103	18–36–123	0.12–0.18–0.27	0.11–0.20–0.33
2	52–81–115	46–66–91	14–43–63	25–44–87	0.14–0.23–0.33	0.14–0.24–0.36
3	50–73–112	48–64–93	20–35–55	21–33–62	0.11–0.22–0.45	0.12–0.20–0.32
4	51–77–97	50–74–97	33–58–92	18–32–50	0.14–0.22–0.37	0.14–0.25–0.39
5	57–82–98	45–71–105	23–61–106	13–33–77	0.13–0.22–0.40	0.10–0.16–0.27
6	62–79–99	63–82–107	32–53–83	66–119–200	0.09–0.16–0.25	0.09–0.15–0.22
7	54–74–97	45–63–83	33–61–94	103–192–321	0.09–0.15–0.23	0.09–0.14–0.23

For every embryo, the distributions of the characteristic diameter *D*, characteristic time-averaged velocity 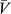, and characteristic length *L* of the vessel segments are respresented by the 25%–50%–75% percentiles for both measurement series T1 and T2.

During development, a network can remodel into a denser network. This was the case for embryo 5: the network at T2 could be described as the network at T1 with additional smaller vessel segments filling up areas between the existing vessel segments. An increase in the number of vessel segments and vessel density (see Table 1) were evident consequences. These observations were also consistent with the changes in other parameter distributions: the measured diameter and velocity distributions showed an increased presence of small blood vessels with corresponding lower velocities, and also an overall shift to shorter vessel segments was measured. This overall decreased vessel length was caused by longer vessel lengths breaking up into multiple smaller vessels due to new branches, and obviously, the new small vessels themselves. A relatively large increase of the vessel density with respect to the vessel occupancy also indicated presence of smaller vessels. Furthermore, these new vessel segments connected the previously existing vessel segments, hence they will have a tendency to be directed away from the main flow direction. The resulting increased spreading in vessel flow direction can be related to the greater standard deviation of the vessel flow direction 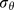 (see Table 4). Embryos 3, 6, and 7 had an approximately similar progression, with the primary difference being an increase in the overall velocities. This resulted from greatly increasing velocity magnitudes in the larger vessels. From a physicogical standpoint, a denser network may be necessary to expand the exchange area for nutrition supply, or, at a later stage, part of these smaller vessels may form the venous network [2].

**Table 4:** Statistics for the modelled vessel network for every embryo.

embryo	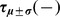		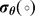		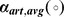		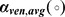		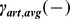		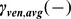	
label (no.)	T1	T2	T1	T2	T1	T2	T1	T2	T1	T2	T1	T2
1	1.10±0.10	1.10±0.11	66	58	93	88	95	86	1.05	0.89	1.25	0.90
2	1.10±0.08	1.12±0.10	56	57	93	83	89	74	0.96	0.94	1.12	0.86
3	1.11±0.08	1.11±0.10	52	59	83	90	84	88	1.03	1.05	0.96	1.12
4	1.11±0.07	1.11±0.09	43	46	80	80	73	81	1.05	1.03	1.18	1.19
5	1.10±0.08	1.09±0.07	37	49	77	82	71	81	1.00	0.96	1.01	0.98
6	1.10±0.08	1.09±0.07	57	53	103	93	103	94	1.11	1.04	1.22	1.04
7	1.09±0.08	1.110.12	64	70	96	94	107	89	1.10	1.81	1.28	1.02

For every embryo, statistics are listed for the collection of vessel segments and branch points for both measurement series T1 and T2: mean 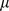 and standard deviation 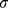 of the tortuosity 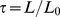, standard deviation of the angle between the vessel segment and the main flow direction 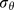, mean arterial branching angle 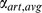, mean venous branching angle 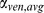, and mean Murray’s law ratio for both arterial and venous branches 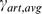 and 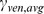. These parameters are illustrated in Figure 7 for clarity.

In contrast to the above general trends, a network can also remodel into a less dense network. This is likely to happen earlier in development: vessels disconnect or merge to make the network more efficient for transport. This was the case for embryos 1 (HH13+ → HH14+) and 4 (HH14 → HH16). Similarly, the measured hemodynamic parameters indicated this process by a decrease of vessel density and even vessel occupancy, and an increase in the overall vessel lengths.

### Development and Structural Parameters

Table 4 lists statistics of several parameters for all measurement series, including vessel tortuosity (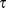), flow direction (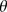), and branching angle (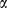). While visually the tortuosity seemed to have changed during development, the statistics of the measured tortuosities did not confirm this. These structural changes could probably better be described as smoothing of the vessel segments with decreasing curvatures. Nevertheless, one notable feature of these statistics was the almost constant mean and standard deviation for all measurement series. Regardless of the stage, the average tortuosity was close to 1.1, which indicates that an extra 10% vessel length is added to the shortest path to obtain the actual vessel length.

The standard deviations of the mean branching angle for each set 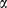 varied between 15 and 38°, with no clear distinction between arterial or venous junctions. The changes in branching angles during development also showed no clear trend, and no considerable differences for this parameter between the arterial and venous branching points in the same measurement region were observed. However, the optimal branching angle, based on the theory for both optimal symmetrical and nonsymmetrical branching should be approximately 90° [41]. This value was closely approximated by this data set. Besides, assuming that a branching angle could have any value between 0° and 180°, with probabilities equally distributed for all possible outcomes, the standard deviation would be 52° (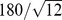, deduced for a uniform distribution). The standard deviations for the different data sets ranged from 15–38°, with an average of 25°. This indicates that the branching angles were not completely random, which would have resulted in a mean and standard deviation of 90°±52°, but were aligned with the flow direction.

The standard deviations corresponding to the mean values of 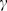 varied between 0.4 and 1.2 (except for the second measurement region for embryo 7). This was more than three times the difference in mean values of 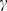 for arterial and venous branches for each measurement region. In addition, the variations between the different measurement regions were found to be minimal, with the exception of the second measurement region for embryo 7. This could be caused by the decreased validity of the two-dimensional approximation of the planar vitelline network. The network extends over a larger part of the curved yolk sac due to growth. The determination of *D* will be affected by these curvature effects. Since 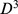 is involved in this law, small errors in diameter extraction have a large effect on the value for 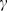. Nevertheless, the theoretical value of unity was in most cases closely approximated by the average value of this parameter. No considerable differences for this parameter between the arterial and venous branching points in the same measurement region were observed.

## Conclusions

A methodology is described where *in vivo* micro-PIV measurements are used to quantify hemodynamics and network structure in relatively large vascular networks. Since the measurement method allows repeated measurements, it can be applied in a network multiple times during development. To demonstrate the possibilities of the method, it has been applied on the yolk sacs of seven chicken embryos at two developmental stages. The developmental stages are in the range of HH stage 13+ to 17+, after remodeling has started and before arteriovenous differentiation has occurred. The measured regions of the vitelline network had an average surface of 13 mm^2^. In total, 3901 vessel segments were characterized. The measured vessel diameters were in the range of 25–500 *μ*m, and the measured time-averaged mean velocity in the range of 1 *μ*m/s – 1 mm/s. With this measurement technique, a large range of flow rates spanning four orders of magnitude were observed. A large variation of flow rates (order of magnitude) can be present for diameters in the same diameter range. This exemplifies that local flow rate measurements (e.g. by micro-PIV) can provide additional information, and can have great benefits compared to studies solely based on imaging. Parameters such as the flow rate and wall shear stress can easily be calculated from the measured parameters, for example to investigate vascular development.

To verify the quality of the data sets, the accuracy of the extracted characteristic vessel diameter and time-averaged velocity were determined by evaluating the relative flow balances in the branch points. Ideally, these relative flow balances should be equal to zero, based on mass conservation. Generally, the relative flow balance distribution could be approximated by a normal distribution around zero, when large outliers were excluded. This analysis showed comparable results for all measurement regions, and an estimated standard deviation of the flow rate of approximately 20%. Furthermore, the two networks that attract attention are the network during early remodeling (HH13+), and the oldest network in this study (HH17+). The network in HH stage 13+ is still a sponge-like plexus instead of a tree-structured network. This complicates defining individual blood vessels, which is likely to influence the relative flow balance. The network in HH stage 17+ starts to extend into the third dimension, which is also likely to influence the relative flow balance. Nevertheless, the quality of the data sets from the seven embryos is comparable, and the data is suitable for further analysis with known accuracy.

We performed a preliminary analysis to highlight the potential of the data set for further research on development. The qualitative observations were compared with the changes in the quantitative parameters. Vascular remodeling was observed in all obtained data sets, but the character of remodeling differed. For example, while some networks became denser, other networks remodeled into a less dense network. Likely, the different locations in the total vitelline network and the developmental stages influence the character of remodeling. Despite the different behavior, the changes in network parameters, such as the vessel diameter, flow velocity, length, and flow direction could be related to the observed structural changes. The agreement of the data sets to design rules for obtaining optimal networks were also investigated. For this, the branching angle and the agreement with Murray’s law were evaluated. For all individual data sets, both the branching angles and the ratios of the involved vessel diameters were comparable and, moreover, closely approximated the optimal values. Further, no notable differences between arterial and venous branches in these parameters were observed.

This study describes a methodology that is capable of quantifying blood flow and network topology at multiple time steps during development. The data set obtained with this method is, to our knowledge, the first available quantitative data set of vascular networks at multiple time steps during development. The data will enable validation of the various vascular network development models. Furthermore, the methodology can also be applied to other model systems, making it a valuable tool to address a wide range of biomedical questions.

## Supporting Information

Figure S1 **Visualizations of the characterized networks for every embryo for both measurement series T1 and T2.**
(PDF)

File S1 **Data on vessel segment characteristics and network topology for every embryo for both measurement series T1 and T2.**
(ZIP)
