# Supplementary material for: Quantification of Blood Flow and Topology in Developing Vascular Networks
Source: PLoS One. 2014 May 13;9(5):e96856. doi: 10.1371/journal.pone.0096856 (PMC4019654; doi:10.1371/journal.pone.0096856)
Supplement: Figure S1 — Visualizations of the characterized networks for every embryo for both measurement series T1 and T2. (PDF) [file pone.0096856.s001.pdf]

Characterized networks of the seven embryos described in the study *‘Quantification of the changing hemodynamics in the developing vascular network of the yolk sac’ - A. Kloosterman, B.P. Hierck, J. Westerweel, and C. Poelma*

For every embryo, the measurement section corresponding to T1 is shown in (a), and the measurement section corresponding to T2 in (b). The embryo number between brackets is the original number used to identify the embryo in the group of all embryos used in this study. All the networks are arterial trees: the flow enters the measurement sections through a large blood vessel, and this vessel branches into smaller vessels. The width of the vessel segments corresponds with the diameters of the vessel segments, and color-coded time-averaged velocity is shown. A non-linear color-coding scale is used to display the velocity, which improves the visibility of the different velocity scales.

Embryo 1 (87) (T1: HH13+, T2: HH14+)

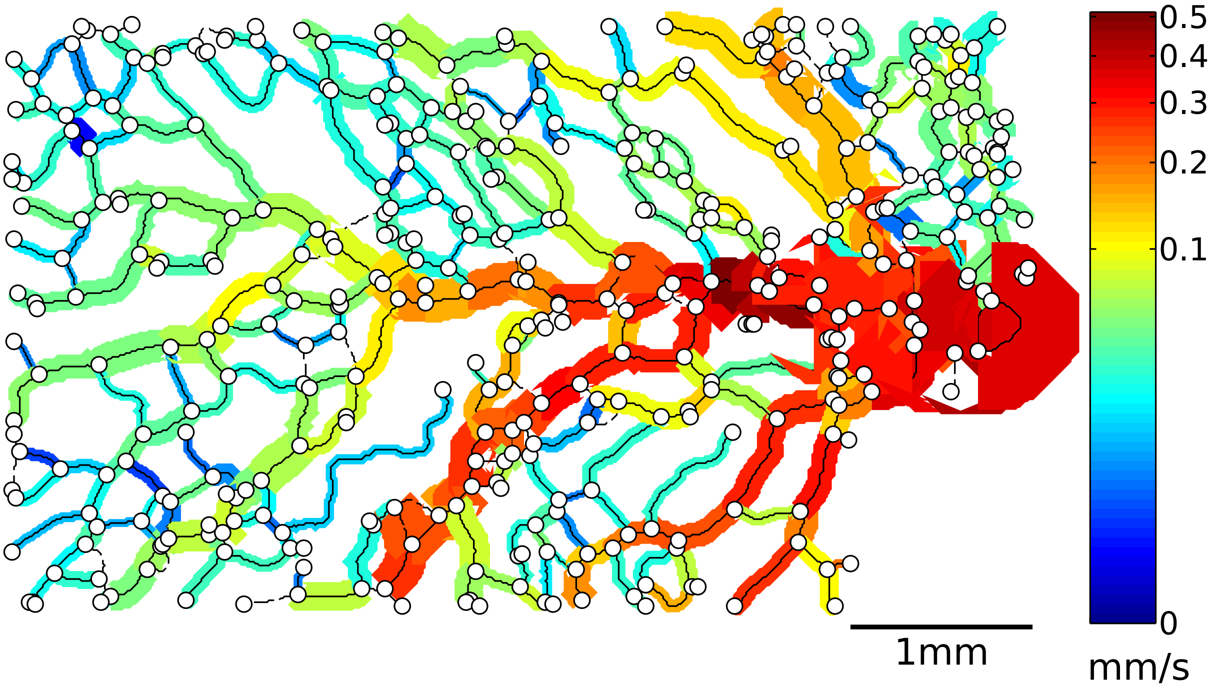

(a)

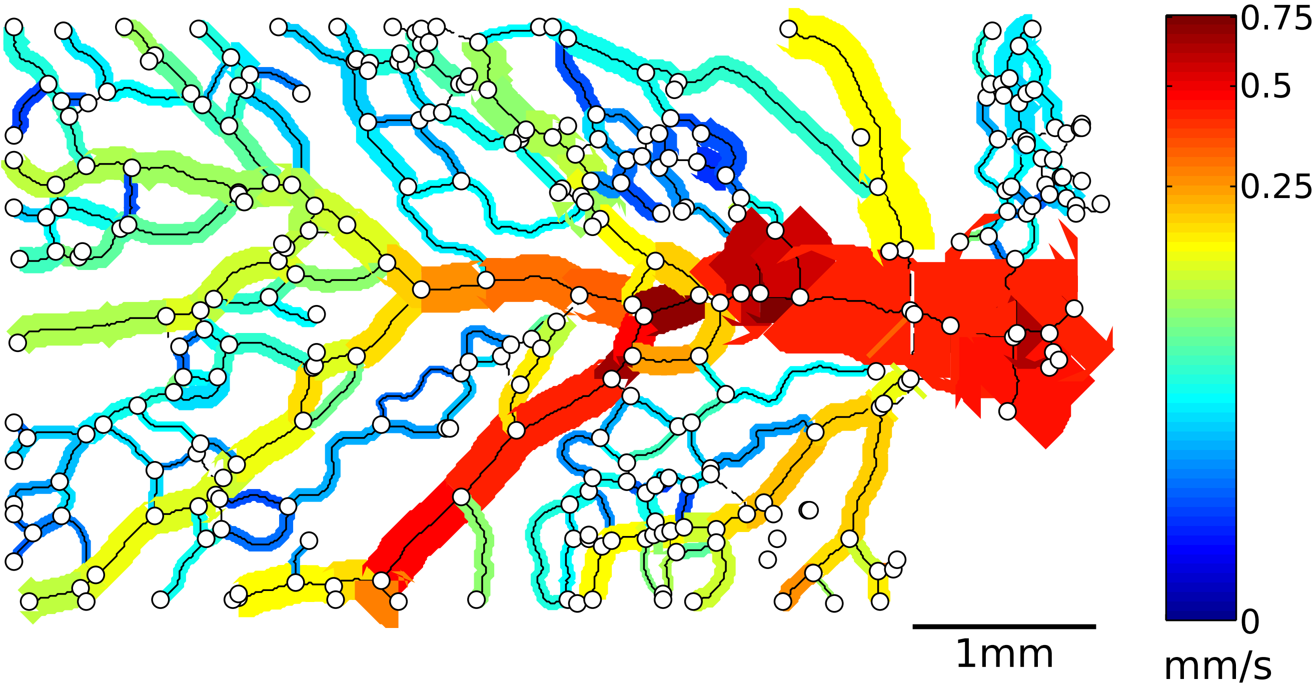

(b)

Embryo 2 (23) (T1: HH14, T2: HH16)

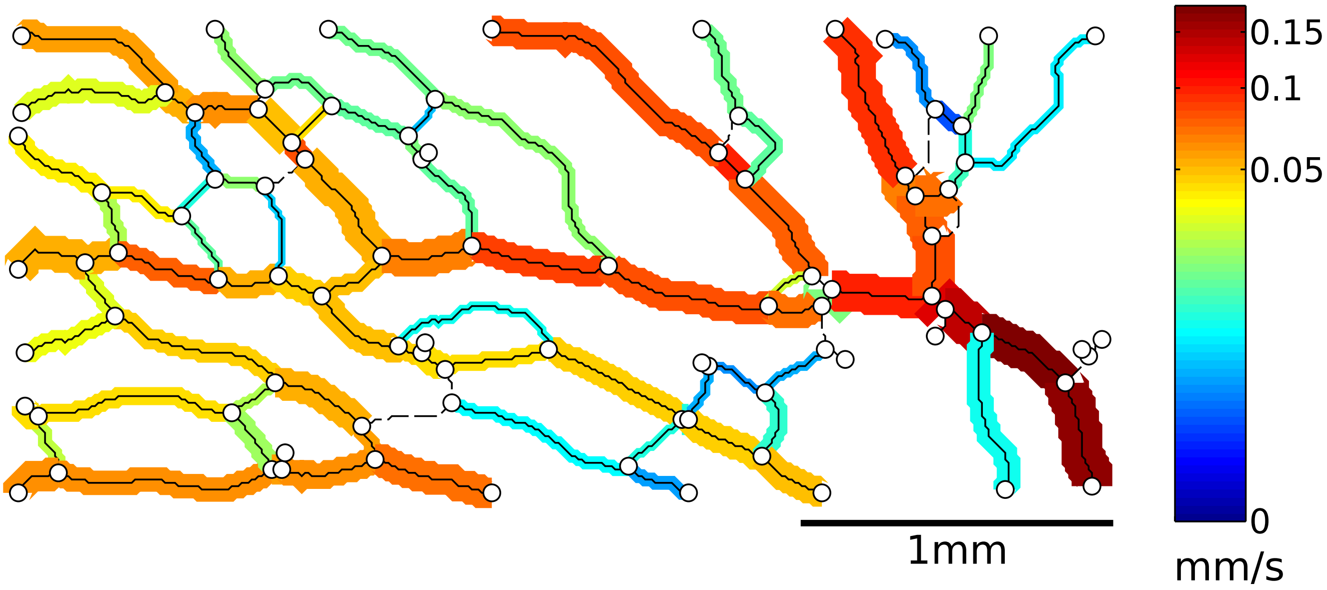

(a)

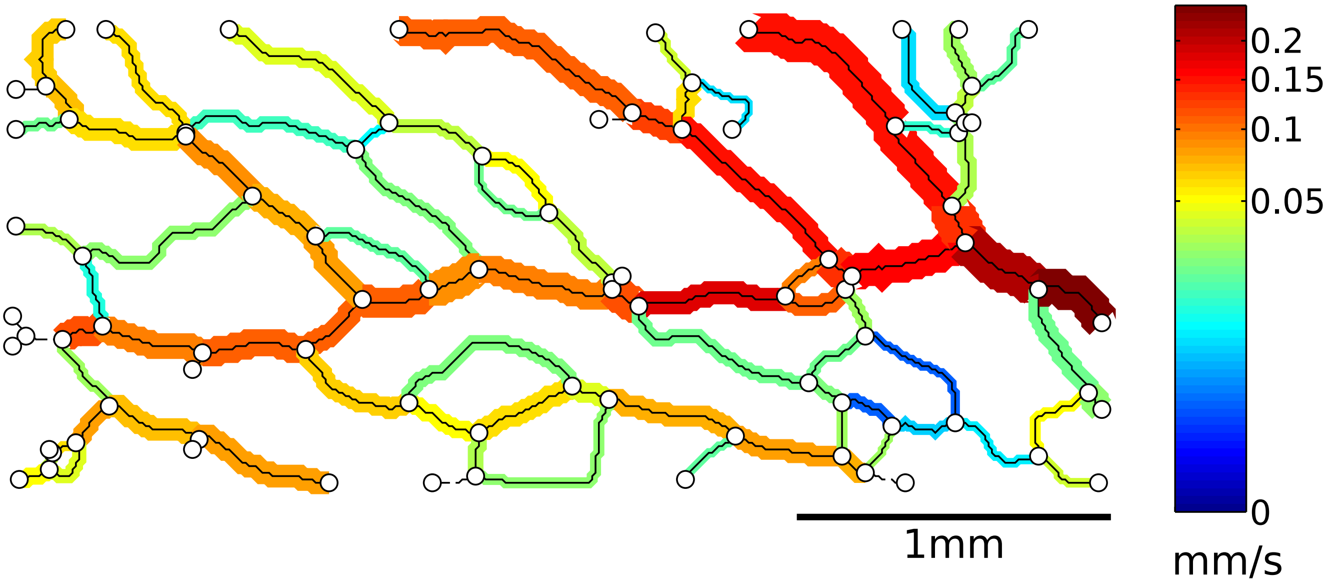

(b)

Embryo 3 (38) (T1: HH14, T2: HH16)

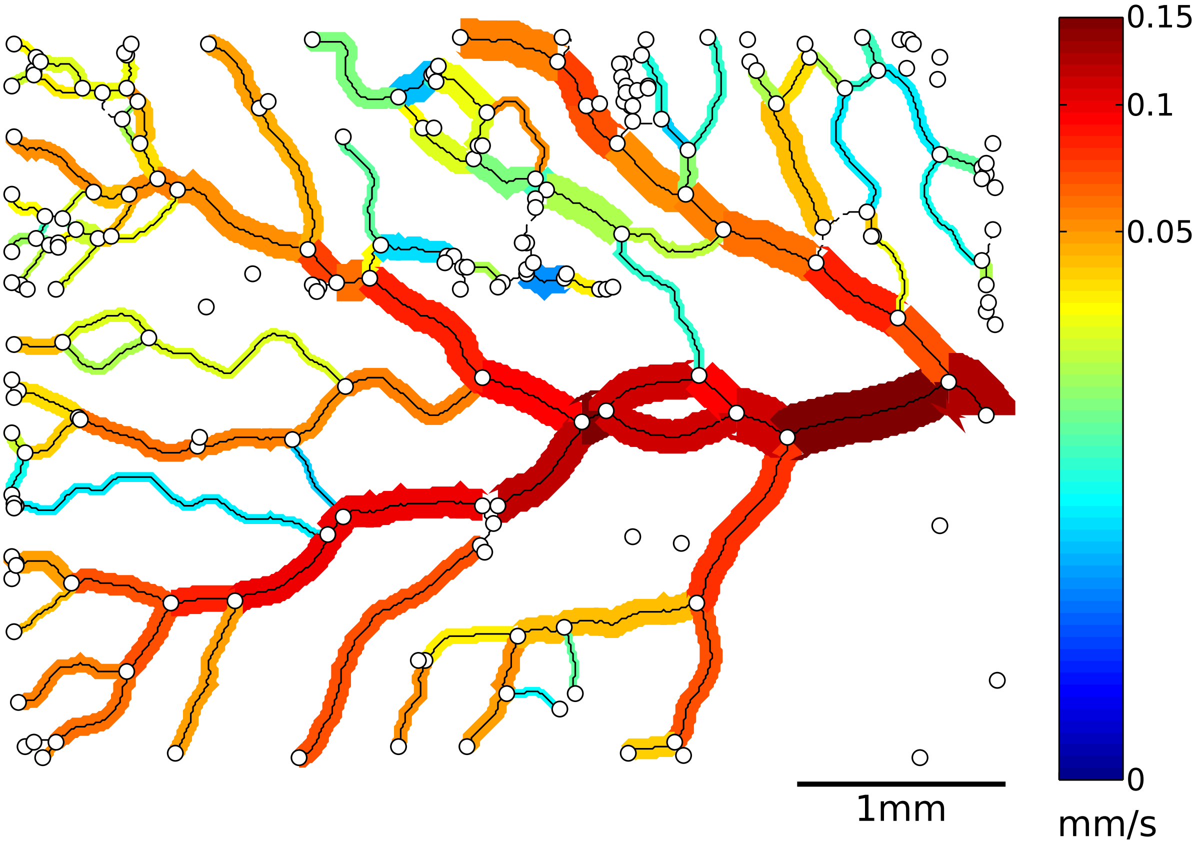

(a)

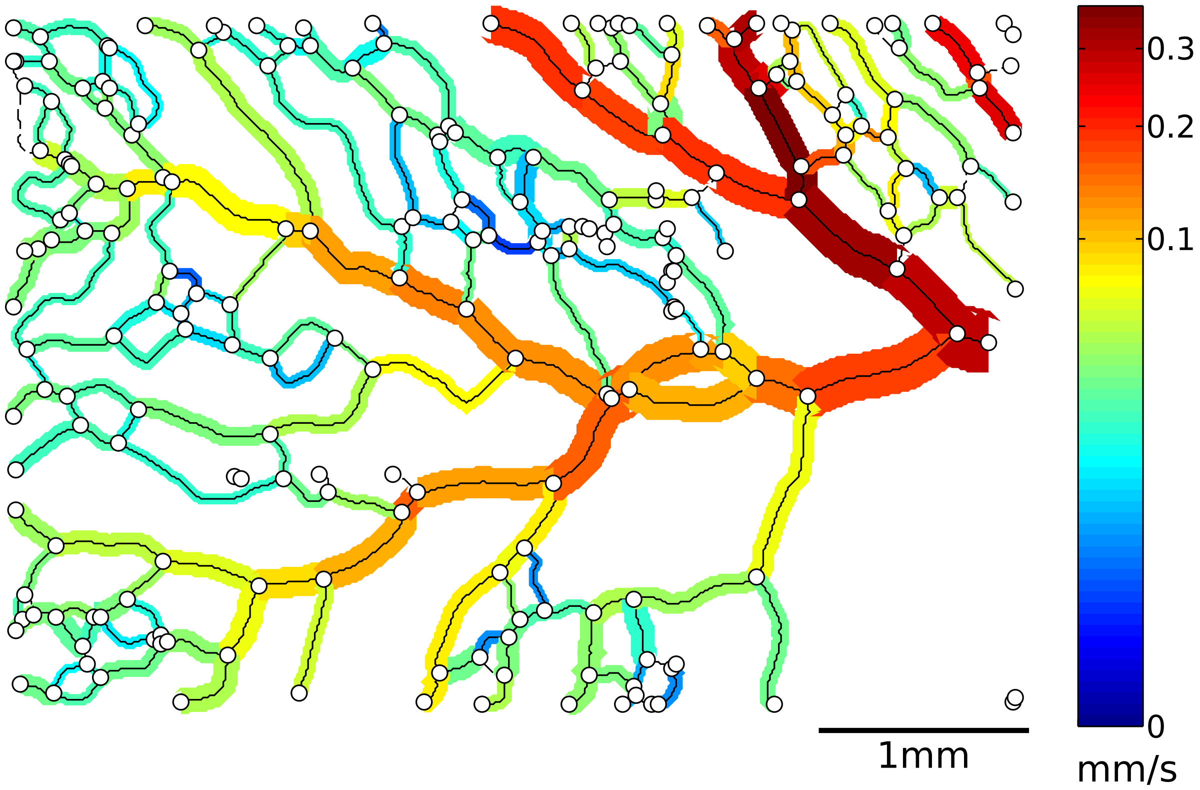

(b)

Embryo 4 (84) (T1: HH14, T2: HH16)

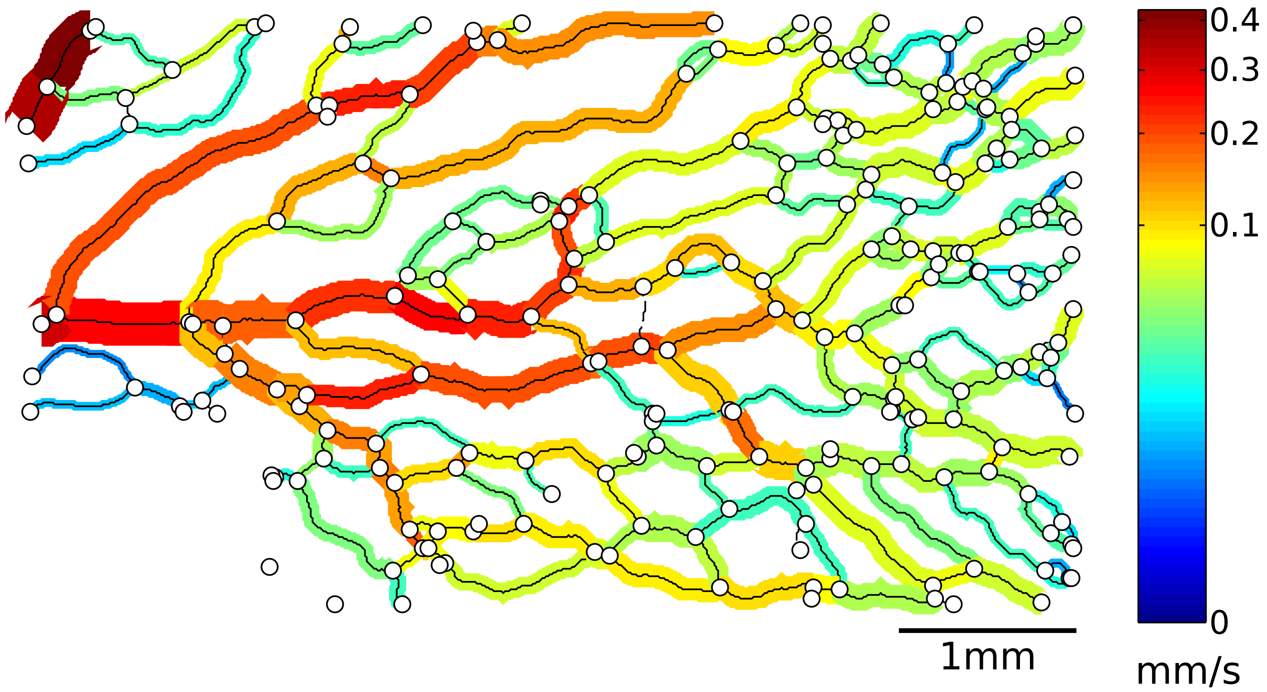

(a)

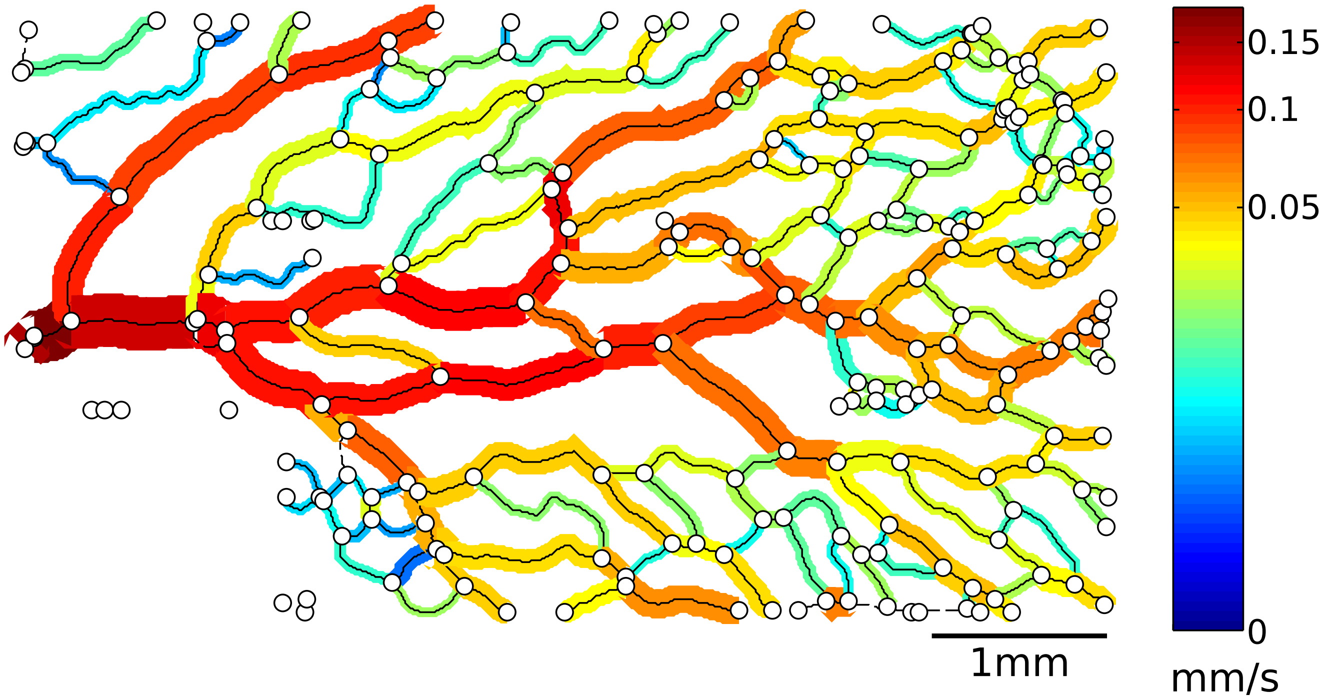

(b)

Embryo 5 (4) (T1: HH15, T2: HH16)

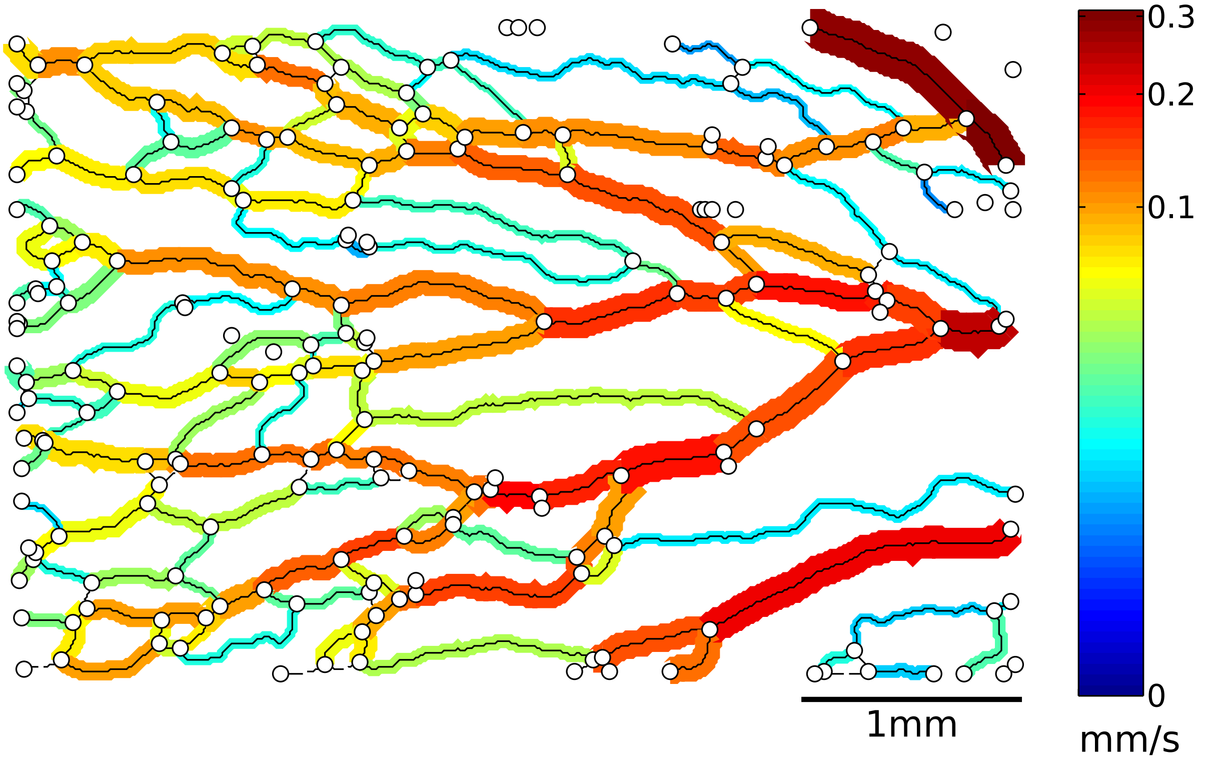

(a)

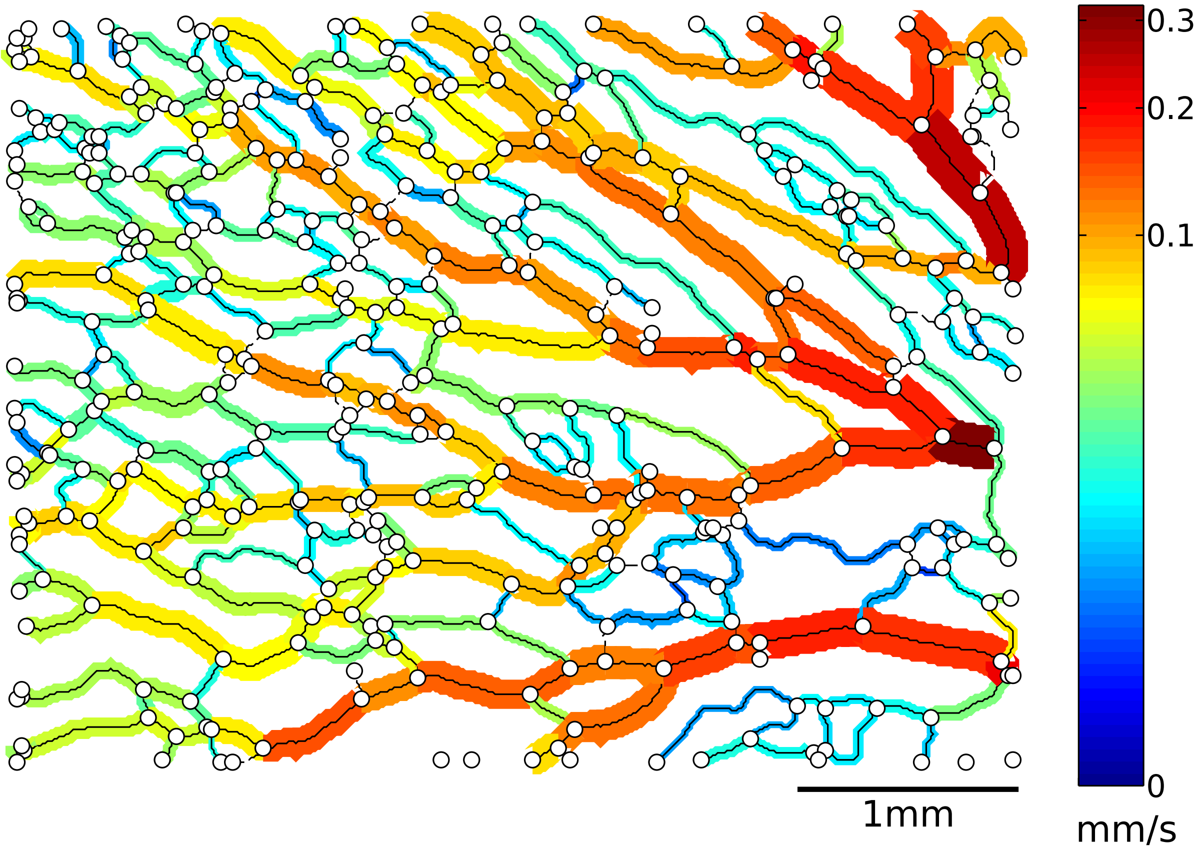

(b)

Embryo 6 (37) (T1: HH15, T2: HH16)

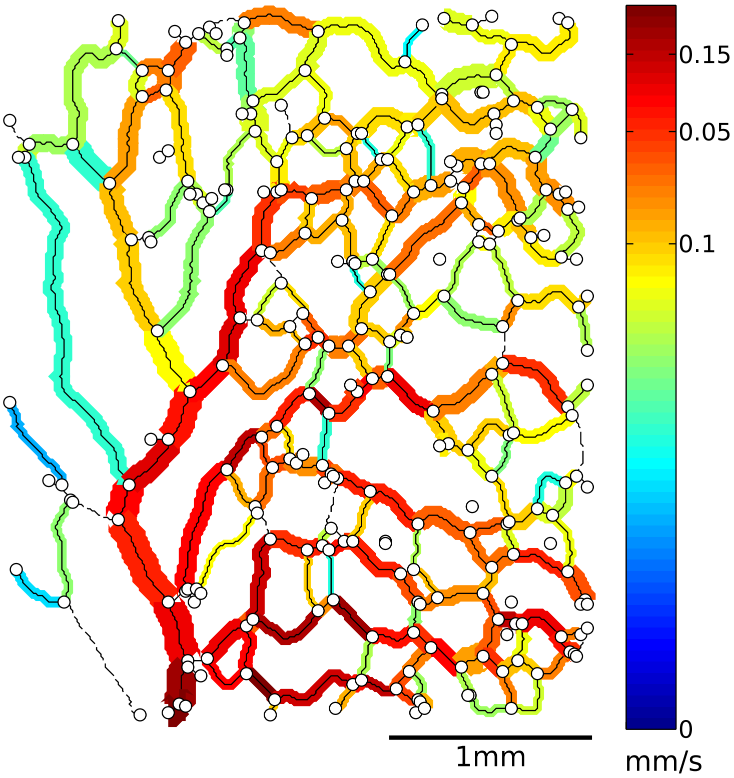

(a)

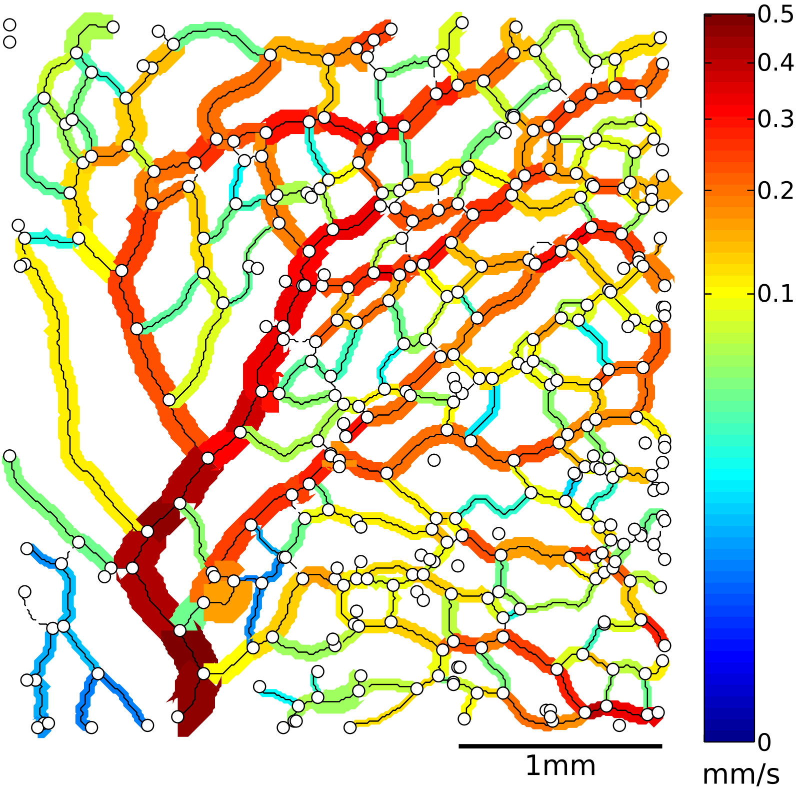

(b)

Embryo 7 (83) (T1: HH16, T2: HH17+)

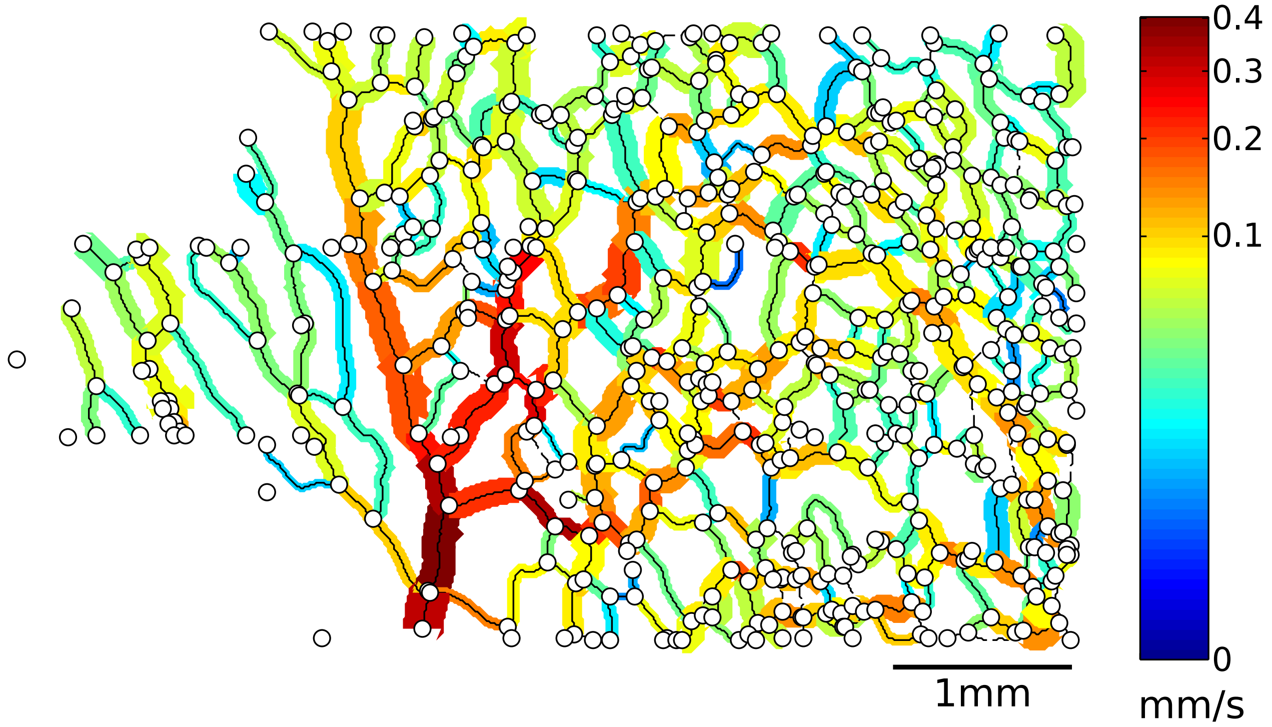

(a)

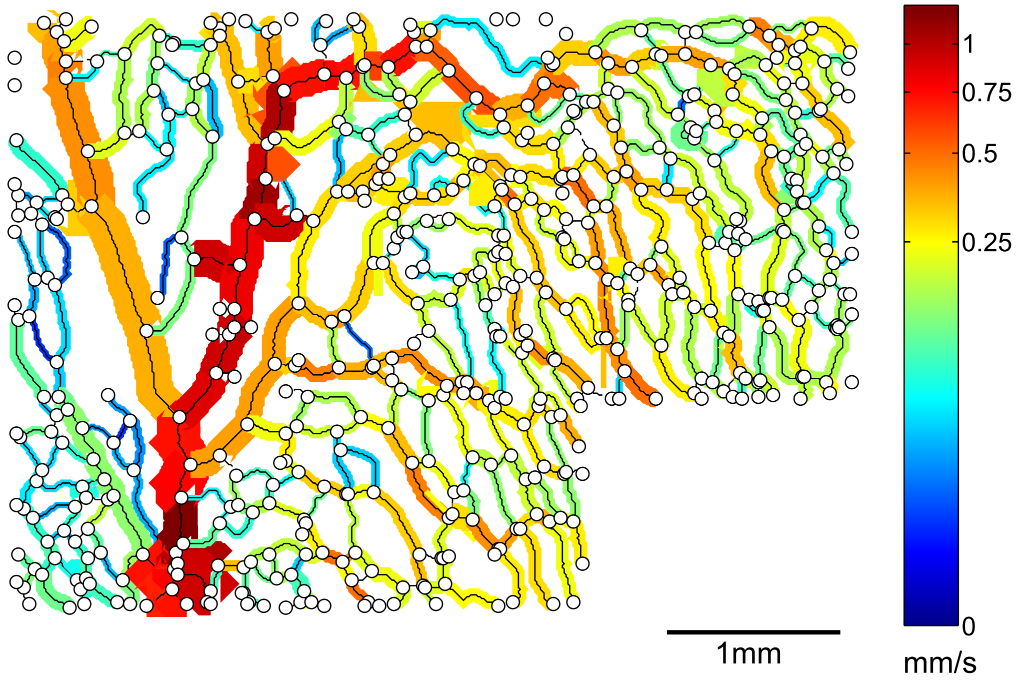

(b)
