# Supplementary material for: Quantification of Blood Flow and Topology in Developing Vascular Networks
Source: PLoS One. 2014 May 13;9(5):e96856. doi: 10.1371/journal.pone.0096856 (PMC4019654; doi:10.1371/journal.pone.0096856)
Supplement: File S1 — Data on vessel segment characteristics and network topology for every embryo for both measurement series T1 and T2. (ZIP) [file pone.0096856.s002.zip › Content_ESM.pdf]

Information on the content of the ESM supporting *'Quantification of the changing hemodynamics in the developing vascular network of the yolk sac'* - *A. Kloosterman, B.P. Hierck, J. Westerweel, and C. Poelma*

This file describes the contents of the files *VesselSegmentCharacteristics_embryoE_Ti.csv* and *NetworkTopology_embryoE_Ti.csv* for every embryo E (E=1,2,3,4,5,6,7), and for both measurement series T_i (i=1,2).

VesselSegmentCharacteristics_embryoE_Ti.csv
This file contains the information about all vessel segments, connecting two branch point (1 and 2). Every row contains the following 11 values, in this order:

n	vessel segment number
x_1	x coordinate of branch point 1 (µm/s)
y_1	y coordinate of branch point 1 (µm/s)
x_2	x coordinate of branch point 2 (µm/s)
y_2	y coordinate of branch point 2 (µm/s)
endpoint	$\begin{cases} 1 & \text{if branch point 2 is an end point,} \\ 0 & \text{if branch point 2 is } \textbf{not} \text{ an end point, but connecting another segment.} \end{cases}$
L	vessel segment length (µm)
$\overline{V}_{\mathrm{CL}}$	time-averaged centerline velocity (µm/s)
D	diameter (µm)
quantified	$\begin{cases} 1 & \text{if a valid } \overline{V}_{\mathrm{CL}} \text{ and } D \text{ have been assigned to this vessel segment,} \\ 0 & \text{if } \textbf{no} \text{ valid } \overline{V}_{\mathrm{CL}} \text{ and } D \text{ have been assigned to this vessel segment,} \\ -1 & \text{if this vessel segment does not represent an actual blood vessel (ghost vessel).} \end{cases}$
flow direction	$\begin{cases} 1 & \text{if blood flows from branch point 1 to branch point 2,} \\ 0 & \text{if no flow direction has been detected,} \\ -1 & \text{if blood flows from branch point 2 to branch point 1,} \\ \text{NaN} & \text{if this vessel segment is a ghost vessel.} \end{cases}$

NetworkTopology_embryoE_Ti.csv
This file contains the locations of the vessel segments. The measurement section is rasterized and the measurement resolution is equal to 10.3 × 10.3 μm^2. This file contains the matrix representing the corresponding measurement section with:
values equal to zero when no vessel centerline is present,
values equal to the vessel segment number when the vessel centerline of the corresponding vessel segment is present,
values equal to -1 when a branch point is present, and
values equal to -2 when an end point is present.
